# Dementia diagnostics in general practitioner care: Do general practitioners have reservations? The findings of a qualitative study in Germany

**DOI:** 10.1007/s10354-019-00722-4

**Published:** 2019-12-06

**Authors:** Julian Wangler, Michael Jansky

**Affiliations:** Centre for General and Geriatric Medicine, University Medical Centre Mainz, Am Pulverturm 13, 55131 Mainz, Germany

**Keywords:** Dementia, General practitioner, Diagnosis, Early detection, Attitudes and perceptions, Demenz, Hausarzt, Diagnose, Früherkennung, Einstellungen und Auffassungen

## Abstract

General practitioner (GP) treatment of dementia is often criticized as being ineffective and not implemented consistently enough. The causes and specific standpoints of GPs have not previously been thoroughly investigated. This paper focuses on the reasons and the criticisms levelled at GPs with regard to diagnosing dementia, and identifies approaches to enable optimization. The analysis is based on 41 semi-structured interviews with GPs in Hesse, Germany, in 2018. During the course of a content analysis, the interviewees’ attitudes and behavioral patterns towards dementia diagnostics were to be analyzed. The results of the study show various challenges and problems of primary care in this field. The majority of the sample showed skepticism and reluctance with regard to the diagnosis of dementia. Six key problem areas were extracted from the interviews, which can be seen as root causes for the distance kept by GPs: 1) early delegation of patients due to role understanding, 2) attitude of pessimism towards dementia, 3) differential diagnosis perceived as an obstacle, 4) insufficient remuneration, 5) fear of patient stigmatization, and 6) lack of application. Some GPs demonstrated personal initiative with the aim of optimizing dementia diagnostics. Three approaches can be derived which could be used to improve the GP-based care of dementia: 1) self-efficacy, 2) differential diagnostics and treatment pathways, and 3) physician–patient communication.

**Electronic supplementary material:**

The online version of this article (10.1007/s10354-019-00722-4) contains supplementary material, which is available to authorized users.

## Abstract (de)

Regelmäßig wird moniert, dass die hausärztliche Demenzdiagnostik und -versorgung nicht effektiv und konsequent genug eingesetzt wird. Die Ursachen hierfür sowie die konkreten Positionen von Hausärzten wurden bislang nur in Ansätzen erforscht. Der Beitrag fokussiert auf die Gründe und Kritik von Hausärzten in Bezug auf die Demenzdiagnostik und leitet Ansatzpunkte für eine hausarztkonforme Optimierung ab. Die Studie basiert auf 41 halbstandardisierten Einzelinterviews mit Hausärzten in Hessen aus dem Jahr 2018. Im Zuge einer Inhaltsanalyse sollten die Einstellungen und Verhaltensmuster der Interviewten in Bezug auf die Demenzdiagnostik analysiert werden. Die Ergebnisse der Studie lassen verschiedene Herausforderungen und Probleme für die Primärversorgung in diesem Bereich erkennen. Beim größten Teil der Stichprobe überwiegen Skepsis und Zurückhaltung in Bezug auf die Demenzdiagnostik. Aus dem Datenmaterial ließen sich 6 grundlegende Problembereiche extrahieren, die als ursächliche Faktoren für die hausärztliche Distanz gesehen werden können: 1) frühzeitiges Delegieren aufgrund des Rollenverständnisses, 2) pessimistische Haltung bei Demenz, 3) differenzialdiagnostische Abklärung als Hürde, 4) mangelnde Honorierung, 5) Sorge vor Patientenstigmatisierung, 6) mangelnde Anwendungsnähe. Bei einer Gruppe von Hausärzten fallen verschiedene Eigeninitiativen zur Optimierung der Demenzdiagnostik auf. Aus der Analyse gehen 3 Ansatzpunkte hervor, auf deren Basis sich die hausarztbasierte Demenzversorgung verbessern ließe: 1) Selbstwirksamkeit, 2) Differenzialdiagnostik und Behandlungspfade, 3) Arzt-Patient-Kommunikation.

## Introduction

Demographic changes are causing a continual increase in the prevalence rate of dementia [1]. This has led to the role of the general practitioner (GP) becoming ever more important with regard to diagnosing and caring for dementia patients. GPs are often the first to be confronted with cognitive changes of their patients or by the patients’ relatives. Consequently, their position is seen as favorable for the early diagnosis, with older patients often having been with the same physician for many years [2, 3] and the general practitioner being aware of these patients’ personal situation [4, 5].

Early diagnosis of cognitive deficits is of utmost importance in order to facilitate early intervention. Even though for the majority of dementias a curative therapeutic perspective does not yet exist, early differential diagnosis could help identify cognitive problems that could be addressed in a causal therapeutic way, especially when they are secondary to underlying causes that can be treated [6, 7]. Even in primary dementia, progression of cognitive symptoms can be attenuated and accompanying behavioral symptoms can better be addressed.

Nevertheless, general practitioners are often subject to criticism for the perceived unsatisfactory care of dementia patients [6–8]. Many complaints are aimed at general practitioners for either not diagnosing dementia or diagnosing it late [9]. Along with an often insufficient overview of current guidelines and treatment options [10–12], this is perceived to stem from insufficient initial and follow-up diagnostics [13]. In this context, inadequate knowledge and unwillingness to apply existing dementia tests is often cited.

Previous study findings do actually show evidence of a more reserved application of diagnostic procedures. This does not only apply to the context of care provision in Germany; studies in other European countries support these findings (for example [14–17]). Additionally, a pilot project for outpatient dementia care showed that the majority of general practitioners prefer to leave the (appropriate) diagnosis to the specialist physicians and concentrate solely on their role as a referring physician [18]. However, these types of mainly quantitative studies often remain open on the question of the causes of this [19].

The findings of some qualitative studies point out that the reluctance of general practitioners to use dementia tests can be attributed to a concentration of corresponding factors. These include time constraints and lack of resources, low expectations of the effectiveness of treatment amplified by a perceived lack of available therapeutic options [20], and fear of patient stigmatization [14, 21, 22].

Abholz and Pentzek [23] point out that there can be a bundling of conflicting goals behind the reservations of general practitioners, especially with regard to diagnostics, where certain aspects of general practice medicine and dementia illnesses come together. On the one hand, the authors explain the general practitioner approach as not being one of combating the condition, but rather a long-term treatment approach to preserve the integrity and independence of the patient. The fact that the expression of suspected dementia or a concrete diagnosis, due to the cognitive and personality-related scope, is seen as a “threat to the self-image” and can quickly lead to a decompensation of the patient and/or their relatives. Consequently, general practitioners often find themselves almost unavoidably in a practically inextricable conflict of objectives [23]. Similarly, the same can be applied to (medication) management or recommendations from the general practitioner for a successful care regime. The reported conflict of objectives makes weighing up necessary and can “have the consequence that aiding the repression of the acceptance of the disease must be supported.”

Despite such insights, only very few studies ask about the more complex correlations relating to the reservations of general practitioners in the field of dementia care. In particular, there is a lack of studies providing general practitioners the opportunity of expressing their standpoint in detail on the challenges of diagnosing, caring for, and treating patients with dementia, in order in this way to obtain a better insight into general practitioners’ reflection, decision, and consideration patterns. It is necessary to carry out studies with a focus on approaches for improved general practitioner care.

The following study aims to determine the predictors for the quality and effectiveness of general practitioner dementia care as holistically as possible. Along with the identification of relevant hurdles, the focus was placed on the general practitioners’ self-perception when identifying and caring for dementia patients, as well as the corresponding attitude and behavioral patterns. Special interest was focused on determining if, from the time of the initial suspicion of cogitative dysfunction affecting everyday life, the general practitioner was motivated to order diagnostics, refer the patient to a specialist or memory outpatient department, and/or carry out diagnostics themselves [18].

## Material and methods

### Research interest, design, and investigation tools

Since there is a lack of reliable studies dealing with GPs’ standpoints, attitudes, and experiences towards dementia diagnostics, there is a need for a broader exploration of this issue. Consequently, a qualitative approach with semi-structured interviews appeared most appropriate. The interview guidelines were developed based on a literature review, especially with the aid of the overview of Pentzek/Abholz [6]. In the course of the first interviews, the instrument was further specified.

The guidelines consist of 33 questions and primarily focus on the following topics: attitude towards the disease of dementia, knowledge of specialist diagnostics, application and assessment of existing testing methods, communication with patients and relatives, practice management, networking with care entities, challenges experienced, and subjective perception of effectiveness (see Electronic Supplementary Material 1).

Using the semi-standardized guidelines, it was possible to get an overview regarding GPs’ superordinate attitudes and behavioral patterns as well as self-perception towards the dementia diagnosis and care of dementia patients. The Department of General Medicine at the University Medical Centre Mainz carried out a total of 41 verbal, semi-structured interviews with general practitioners in the State of Hesse, Germany, between February and July 2018. The duration of the interview was between 45 and 90 min. The interviews were carried out by both authors who have a lot of experience in qualitative research.

Due to the use of the guidelines and intensive coordination between the authors prior to the start of the interviews, it was possible to ensure that the interviews were conducted in a similar manner. The interviews did not involve any personal experiences or positions of the authors. The openness of the interviews also allowed new aspects to be captured. The interviewees were given the opportunity to give their assessment feedback, which some of them did, but this was not recorded.

### Research population and recruitment

The recruitment of the general practitioners interviewed took place using the predefined quota characteristics listed in Table 1. Using a quota system presents advantages in cases of unwillingness, since a study participant can be replaced in a targeted manner. A total of 48 physicians were contacted via telephone or e‑mail, with a total of 41 interviews finally being carried out. Additionally, attention was focused on including a wide geographical distribution of individual practices throughout the state.

**Table 1:** Sociodemographic characteristics of the sample

Sociodemographics (*N* = 41)	
Type of practice	54% (22) joint practice, 46% (19) single practice
Practice location	44% (18) rural community/small town, 24% (10) medium-sized town 32% (13) large city
Status	78% (32) practice owner, 22% (9) salaried physician
Age	Ø 52 years
Sex	59% (24) male, 41% (17) female
Proportion of older patients^a^	39% (16) on average, 34% (14) higher, 27% (11) lower
Previous knowledge/qualifications in the field of dementia	17% (7) additional training, 10% (4) regular participation in quality circles, 10% (4) further geriatric training

^a^The participants were asked for their own estimate of whether the proportion of patients older than 65 was higher, lower, or the same as the average for general practices in Hesse

### Data analysis

The theoretical saturation was reached, so that further interviews were not required. Interviews were transcribed verbatim. Each transcript was double-checked for inaccuracies. The analysis of the transcripts recorded during all the interviews was carried out with the use of MAXQDA software (VERBI GmbH, Berlin, Germany). The data were analyzed according to the method of qualitative content analysis based on Mayring [24]. As part of the analysis, a category system was created, which was repeatedly tested and modified as the analysis progressed. In this way it was possible to condense and systematize differences and similarities in the data in the form of arguments or problematic patterns. The created category system is based on the priorities set in the guidelines.

Table 1 provides an overview of the participating samples.

Selected results of the study are presented below. The findings relate to the interviewed general practitioners’ widespread critical reservations about dementia diagnostics towards the diagnosis and care of dementia patients. At the center of the presentation of the findings is the question on the reasons behind this reserved attitude. Subjective efficacy perception and the self-perception of the general practitioners both play an important role in this. Finally, suggestions for improved approaches will be derived from the collected data.

## Results

A clear majority of the interviewed general practitioners have a distanced or negative attitude toward existing dementia tests. 30 of the surveyed general practitioners have a clearly defined reserved or critical standpoint on dementia diagnostics.

**Fig. 1:**
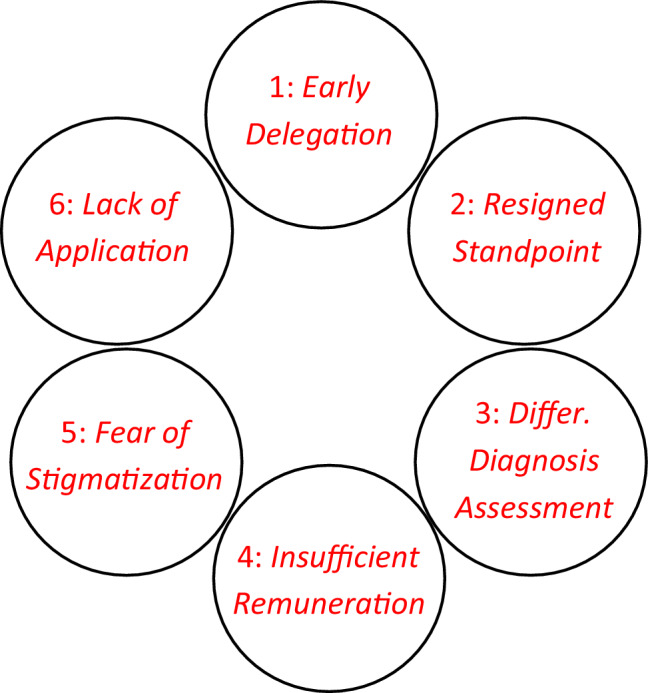
General practitioners’ attitudes towards dementia diagnostics—key problem areas

Depending on the interviewed physician’s standpoint on diagnostics, there was a direct correlation of consequences for the care of dementia patients in their own practice: various behavioral patterns and strategies for dealing with dementia patients were observed. While some GPs tend to refer patients at an early stage to specialist physicians and other healthcare providers, others devised their own internal initiatives in the form of good-practice approaches, and they used this to augment or revamp the dementia care in their own practice. Fig. 1 shows the key problem areas that were identified in the course of the interviews.

### Early delegation as the result of the understanding of one’s role

A group of those interviewed, as part of their own perception of the role of a general practitioner, find dementia tests to be no longer required. These general practitioners do not see themselves as being responsible for the clarifying diagnosis and care of dementia patients but find that this responsibility lies with specialist physicians.

*I expect the treatment of dementia patients to be carried out primarily by neurologists. They are the ones responsible.* (male)

Their own role is perceived primarily as that of referring the patient as quickly as possible to neurologists, (geronto)psychiatrists, or the outpatient memory department. This often takes place based only on a general and unspecified suspicion with no further diagnostics carried out. This is because, in principle, dementia tests at their own practice are seen as unnecessary, so there has been a notable reduction or complete discontinuation of diagnostics in these practices.

### Pessimistic attitude towards dementia

Another group of GPs also considers it unnecessary to provide dementia diagnostics in their own practice. The high-level of reluctance arises from an extremely low perception of efficacy and a feeling of a lack of suitable treatment options in the field of caring for dementia patients.

Many physicians view this self-efficacy exclusively from the medication-based treatment standpoint and rarely consider other forms of general practitioner support. Since it is assumed that the treatment and care of dementia per se offers no hope of healing, no advantage is seen in early diagnosis of cognitive impairment.

*We are healers, and when we have to deal with something like dementia it affects our self-perception. […] There is no point, we can’t do anything. It is therefore better to diagnose it at a later stage. That way you don’t have it in front of you all the time without being able to do anything, you know?* (male)

Due to their pessimistic attitude, those interviewed have often discontinued or reduced the dementia diagnostics (e.g., clock-drawing test) on offer at their practices. Usually, with an undifferentiated dementia diagnosis, e.g., based on reports from relatives, the patients are quickly referred to specialist physicians.

### Differential diagnostic investigation perceived as an obstacle

A proportion of those interviewed justified their reservations in using dementia diagnostics by the difficult overall context of a differential diagnostic investigation. It was articulated that the necessary exclusion diagnostics were very difficult to perform correctly under the everyday time constraints. Those interviewed also stated that it was not possible to differentiate normal degenerative age progression definitively from age-related depression, dementia, or Alzheimer.

*We do not have the confidence to carry out the differential and exclusionary diagnostics in their full extent and differentiation.* (male)

In light of these concerns, it is evident that the availability of certain types of dementia tests alone is an inadequate instrument to clarify the cognitive status of an affected patient.

*What is the point of one single test when the overall complex “clarification” is so tricky?* (female)

The general practitioners in this group tended to immediately refer patients to the specialist physician and delegate possible dementia patients.

### Insufficient remuneration

There are interviewees who do not show a pessimistic attitude, but nevertheless a very reserved standpoint. They justify their very marginal application of dementia diagnostics with the argument that the increased usage of tests and dementia-related consultation and care services could leave them at an economic disadvantage.

*This is a loss-making business for a general practitioner. In its current state, it is simply an economic ball and chain.* (male)

According to those interviewed, the remuneration in this field is so poor that it is in no way proportionate to the amount of effort and the challenges involved. Several of the physicians spoke of a lack of recognition.

*It has to do with appreciation. This type of appreciation must have its place within the system, otherwise the system is not correct.* (male)

### Fear of stigmatizing of the patient

Part of the GPs interviewed connect their reservations about the diagnostics to the physician–patient relationship. Those interviewed report that dealing with patients becomes difficult, e.g., when suggesting the patient undergoes a dementia test or when formulating a suspected diagnosis or diagnosis. This is often blamed on the existing test method which puts the patient in an exam-like situation and conveys a latent feeling of stigmatization.

According to many of those interviewed, this leads to the patient refusing to cooperate further, either during or following the test, because they do not want to admit mental deterioration. This causes the patients to become defensive, depressive, aggressive, or to withdraw from society. This behavior is due to the underlying fear of being robbed of one’s own powers of decision.

Individual physicians admitted that they have put poor test results down to the patient’s mood on that specific day or to personality traits, because they were apprehensive of a negative reaction from the patient. Since the tests are not subtle enough, general practitioners have no effective instrument available enabling them to conduct a differentiated classification.

*I will scare my patients away as soon as I start to carry out tests with them. The patients feel under pressure or begin to panic and finally they decide to stay away from the practice entirely. Is that the desired effect? No, not at all.* (male)

In addition, a conflict of objectives is addressed when it comes to confronting patients early and, consequently, with a suspected or concrete diagnosis of dementia.

*As a general practitioner, it is in your interest for the patient to be in a psychologically and socially stable state. Therefore, in some cases, you accept situations even when you know it is very likely that there is an underlying issue. You begin to wonder when is the correct time to present a patient with such a negative perspective—telling them that they have been diagnosed with dementia. […] There is a danger that, as a physician, you end up in a downward spiral of inaction.* (female)

### Lack of application

Some GPs state that, from their perspective and experience, the tests are not sufficiently applicable. Three reasons were given for this. Firstly, the fundamental orientation of the questions is viewed with skepticism, as they are deemed to be far from the reality of life for older people and therefore do not offer valid and relevant indicators.

*The problem with the tests is that they are not applicable to everyday situations. They don’t contain questions such as “Do you cook for yourself?”, “Have you ever forgotten to turn the stove off?”* (male)

Secondly, the established tests were criticized for failing to stop the initial signs of dementia. This in turn is explained by their content aspects, as there are insufficient reliable indicators for the identification of low-to-moderate-grade dementia.

Thirdly, doubt is cast on the validity of dementia tests. Since the current diagnostics place the patient in a none-too-subtle testing situation when faced with the impact of a potential dementia diagnosis, a consistent high level of reliability cannot be expected from the tests.

*Since the patients are already extremely agitated when I tell them I want to carry out a dementia test, then one can safely doubt the accuracy of the results. The tests simply are not sufficiently patient compatible.* (female)

### Initiative to improve existing dementia tests

Due to the reservations about the practicability and user-friendliness of existing dementia tests, it was noted that some of the physicians interviewed have thought about ways to complement dementia diagnostics through their own activities. This group understands the general practitioner as being the main point of reference for the identification, care, and therapy of dementia patients. Provided that new, unconventional approaches can be applied, significant development and increased efficacy is seen to be possible. The most important good-practice activities are summarized below:

- Systematic screening of older patients going beyond cases with concrete suspicion and consequent check-ups (identifiable willingness, with the introduction of preventive screening more extensively than recommended in current dementia guidelines [25, 26])
- Systematic inclusion of the patient’s relatives as an additional screening instrument and for support (discussions at regular intervals)
- Independent augmentation and/or modification of existing tests (partially based on the sharing of experiences with other general practitioners), to improve applicability and patient-friendliness
- Development and implementation of additional early-recognition indicators (e.g., filling out “mood questionnaires” in a specially designated and decorated part of the practice, subtle test questions presented by staff)
- Integration of practice staff into the dementia early-recognition process (regular further training, sensitization for dementia symptoms, improvement of practice organization, continual contact via conversations with patients and their relatives, application of indicator questions, active participation of staff when designing the early-recognition procedures)

An example of a solution created by the physician and the staff is that if the staff notice behavioral abnormalities in older patients (e.g., repeated requesting of a prescription, no-show for appointments), then this should be noted in the patient’s file. Additionally, opportunities for cooperation should be pursued, for example, care support entities, dementia networks, physiotherapists, and specialist physicians.

## Discussion

### Main findings and interpretation

The findings show that general practitioners are highly critical of current dementia diagnostics. This includes reservations about a consequent application of the test methods. The latter are not deemed to be optimal for the general practitioner setting and its specific conditions. This refers to the questions of everyday applicability and user-friendliness which, according to many of those interviewed, are not sufficiently addressed by the current methods.

Additionally, significant elements of uncertainty were recorded. This correlates to the findings published in specialist literature which state that, despite general practitioners’ excellent position for the early identification of cognitive changes in patients, many barriers remain [6, 7, 21, 27]. The grouping of the 41 interviews showed that certain patterns of problems accumulate, and these then act as obstacles to the provision of dementia diagnostics by the general practitioner:

- *Self-efficacy:* Many of the interviewed general practitioners report that insufficient therapeutic relevance is what leads to a perception of low subjective self-efficacy [28]. This leads to the general practitioners doubting the value of the dementia diagnostics [6].
- *Differential diagnostics and treatment pathways: *In the view of a segment of those asked, differential diagnostic clarification is a challenge in everyday practice. Perceived uncertainties in differentiating dementia from other forms of cognitive impairment [29] are amplified by the ambiguities in the therapeutic process.
- *Physician–patient communication:* The potential for role definition conflict when dealing with dementia patients means that the general practitioner tries to avoid tension and psychosocial decompensation of the patient and their relatives. This means that dementia tests are not applied consequently, but rather on a case-by-case basis; the test results are regarded in a variety of ways.
- *Remuneration:* From the standpoint of a segment of those interviewed, general practitioners do not have the necessary incentives to apply dementia diagnostics consequently. Realistic fears of a performance audit mean that indicated anti-dementia medication is often not prescribed [30].

### Strengths and limitations

The qualitative surveying of general practitioners has several limitations, which should be correspondingly reflected upon:

- The study is based on a small sample size, so that the findings have to be considered as non-representative.
- The study has a regional recruitment focus.
- The aim of the study was to give a broad overview of GPs’ attitudes and behavioral patterns. Therefore, it was not possible to address certain aspects in more depth. For example, the study could have addressed the ethical questions associated with revealing early, even preclinical diagnosis of dementia to the patient before he/she is even affected by cognitive functional problems. This remains an issue for future research.
- It cannot be ruled out that greater numbers of general practitioners took part who already had an interest in the topic of dementia (selection bias caused by previous knowledge)
- The study was conducted in the German general practitioner care context having its own specifics; generalizations to the European level are not possible in every aspect

However, the chosen methodical approach and the heterogenic sample allow for claims to be made which are applicable to the full spectrum of general practitioners.

## Conclusion

The results confirm the previous research and suggest that general practitioners are reserved in the application of dementia diagnostics due to perceived or feared risks, uncertainties, and burdens. This has a direct effect on the efficacy of the (early) recognition and timely care of patients.

General practitioners should be encouraged to recognize the value of the earliest possible identification of dementia, not only regarding therapeutic intervention, but also to ensure the successful implementation of care for the patient and stabilization of the relatives. In addition, it would be useful to develop a general practitioner-suitable diagnosis and treatment algorithm which would help support general practitioners in the diagnosis, care, and treatment of dementia patients. Moreover, stabilizing strategies in conversations with patients and relatives are crucial competencies for the diagnosis of dementia and continued patient care [18]. This should lead to an expansion of expertise at the general practitioner level.

In a number of those interviewed, the great potential of general practitioner care is visible. Particularly trendsetting are attempts to augment the existing tests with independent innovative early-recognition indicators, while having staff actively participate in the recognition. Cooperation between general practitioners and other care provider entities should be further strengthened in order to create care advantages [31, 32]. If patients and their relatives are put in contact with regional advice and care networks [33] early enough, then, for example, the risk of relatives acting as caregivers [34] and suffering from burnout can be reduced.

## Caption Electronic Supplementary Material

Interview guidelines
