# Supplementary material for: Dementia diagnostics in general practitioner care: Do general practitioners have reservations? The findings of a qualitative study in Germany
Source: Wien Med Wochenschr. 2019 Dec 6;170(9):230–7. doi: 10.1007/s10354-019-00722-4 (PMC7272384; doi:10.1007/s10354-019-00722-4)
Supplement: Supplementary file 1 — Interview guidelines [file 10354_2019_722_MOESM1_ESM.docx]

**Interview Guidelines**

- From your experience, what are the most common signs of incipient dementia?
- What advantages do you see in recognizing dementia as early as possible? What benefit does early detection have for the well-being and quality of life of the patient?
- What options does the general practitioner have to recognize (early) dementia?
- Do you use diagnostic tests in your practice?
- Which tests do you perform regularly? (e.g., MMST, DemTect, Clock Drawing Test, MoCA)
- From your experience, which test is well suited?
- What strengths and weaknesses do the tests have?
- How important is it for you to clarify the suspicion of dementia with your own diagnosis? Why?
- In your opinion, is it better to leave the diagnosis to a specialist? Why?
- How common is it for you to refer to a specialist physician or an outpatient clinic due to suspected dementia? To which specialist do you transfer your patients ([geronto]psychiatrist, neurologist)?
- Under what conditions would you express a suspicion of (incipient) dementia, under what conditions would you refrain from it?
- Do you also treat patients with dementia or do you usually leave this to a specialist? (e.g., anti-dementia drugs)
- To what extent do you make use of the dementia guideline (S3)?
- How useful is the guideline from your point of view?
- How easy or difficult is it for the general practitioner to identify or diagnose (incipient) dementia? What are the reasons for this? What do you consider to be the biggest challenges in detecting dementia in general practice?
- Time and again, one hears and reads that in many general practices the dementia of (long-term) patients remains unrecognized for a long time and is often being diagnosed only at an advanced state. Why do you think that is?
- To what extent do general practitioners see themselves confronted with a conflict of objectives when it comes to diagnosing or treating dementia patients (e.g., disease control vs. autonomy of the patient)? What can you report from your own experience?
- Which aspects of dementia diagnosis and care do you experience as challenging? Why?
- How competent do you feel when it comes to early detection of dementia in general? Where do you see your strengths, where do you see your weaknesses?
- Have you ever been in a situation where you deliberately postponed a dementia diagnosis because you were concerned that a diagnosis would have a very negative impact on your patient and/or his/her relative(s)? How often do such situations occur?
- If you think of the patients you have diagnosed with dementia: Did you find it easy or difficult to let them know? Why? How did the patients react?
- Have you ever had inhibitions or uncertainties of informing a patient that you suspect him/her of having dementia? Why?
- What significance do relatives have for dementia detection?
- How do you assess the collaboration of general practitioners and specialists, such as (geronto)psychiatrists or neurologists, when it comes to the (early) detection of dementia? Where do you see problems and potential for improvement?
- To what extent are you familiar with the regional and local care structures specifically for the care and treatment of dementia patients? (e.g., care support entities, dementia networks, physiotherapists, specialist physicians, memory clinic)
- To what extent do you cooperate with specific facilities and services for the care, consultation and care of dementia patients?
- Would it make sense to cooperate with other institutions and services? What could you imagine?
- What measures, do you think, would help GPs to diagnose dementia sooner? What should be changed or improved? (e.g., regular dementia screening of elderly patients)
- It is sometimes suggested that the early detection of dementia would be more successful if there were specially trained members of the practice staff who are experienced in the subject and actively assist doctors in caring for dementia patients. Do you think that this could significantly improve the early detection of dementia patients?
- Are there certain members of your practice staff who are experienced on the topic of dementia as well as in caring for dementia patients and have undergone trainings?
- What do they contribute to the (early) detection of dementia? Why?
- Do you feel there is a need for further training on the topic of diagnosing and treating dementia?
- Did you attend training activities on the diagnosis, monitoring and treatment of dementia? Which trainings exactly? How satisfied were you with these trainings?
